# A Major Intestinal Catabolite of Quercetin Glycosides, 3-Hydroxyphenylacetic Acid, Protects the Hepatocytes from the Acetaldehyde-Induced Cytotoxicity through the Enhancement of the Total Aldehyde Dehydrogenase Activity

**DOI:** 10.3390/ijms23031762

**Published:** 2022-02-03

**Authors:** Yujia Liu, Takumi Myojin, Kexin Li, Ayuki Kurita, Masayuki Seto, Ayano Motoyama, Xiaoyang Liu, Ayano Satoh, Shintaro Munemasa, Yoshiyuki Murata, Toshiyuki Nakamura, Yoshimasa Nakamura

**Affiliations:** 1 School of Biological Engineering, Dalian Polytechnic University, Dalian 116034, China; lyj32731@126.com; 2 School of Food Science and Technology, Dalian Polytechnic University, Dalian 116034, China; LiKexin0903@hotmail.com (K.L.); liuxiaoyang0213@126.com (X.L.); 3 Graduate School of Environmental and Life Science, Okayama University, Okayama 700-8530, Japan; p5qc6ubd@s.okayama-u.ac.jp (T.M.); pxvz3vwr@s.okayama-u.ac.jp (A.K.); pl8o0zi3@s.okayama-u.ac.jp (M.S.); ayano1122@s.okayama-u.ac.jp (A.M.); smunemasa@okayama-u.ac.jp (S.M.); muta@cc.okayama-u.ac.jp (Y.M.); t-nakamura@okayama-u.ac.jp (T.N.); 4 Graduate School of Interdisciplinary Science and Engineering in Health Systems, Okayama University, Okayama 700-8530, Japan; ayano113@cc.okayama-u.ac.jp

**Keywords:** 3-hydroxyphenylacetic acid, aldehyde dehydrogenase, quercetin metabolites, aryl hydrocarbon receptor, acetaldehyde

## Abstract

Aldehyde dehydrogenases (ALDHs) are the major enzyme superfamily for the aldehyde metabolism. Since the ALDH polymorphism leads to the accumulation of acetaldehyde, we considered that the enhancement of the liver ALDH activity by certain food ingredients could help prevent alcohol-induced chronic diseases. Here, we evaluated the modulating effects of 3-hydroxyphenylacetic acid (OPAC), the major metabolite of quercetin glycosides, on the ALDH activity and acetaldehyde-induced cytotoxicity in the cultured cell models. OPAC significantly enhanced the total ALDH activity not only in mouse hepatoma Hepa1c1c7 cells, but also in human hepatoma HepG2 cells. OPAC significantly increased not only the nuclear level of aryl hydrocarbon receptor (AhR), but also the AhR-dependent reporter gene expression, though not the nuclear factor erythroid-2-related factor 2 (Nrf2)-dependent one. The pretreatment of OPAC at the concentration required for the ALDH upregulation completely inhibited the acetaldehyde-induced cytotoxicity. Silencing AhR impaired the resistant effect of OPAC against acetaldehyde. These results strongly suggested that OPAC protects the cells from the acetaldehyde-induced cytotoxicity, mainly through the AhR-dependent and Nrf2-independent enhancement of the total ALDH activity. Our findings suggest that OPAC has a protective potential in hepatocyte models and could offer a new preventive possibility of quercetin glycosides for targeting alcohol-induced chronic diseases.

## 1. Introduction

Acetaldehyde is the first ethanol metabolite that might mediate various types of ethanol-induced abnormal behaviors and chronic diseases, such as liver diseases, cardiovascular diseases, carcinogenesis, neuropsychic disorders, and addiction [1]. Due to its electrophilic nature, acetaldehyde can directly modify proteins, lipids, and DNA to form covalent conjugates [2]. These conjugates might influence cellular homeostasis and survival through protein structure alteration and/or DNA damage. Aldehyde dehydrogenases (ALDHs) are one of the major enzyme superfamilies that play a key role in the metabolism of aldehydes [3]. Among the 19 ALDH family enzymes, ALDH class-2 (ALDH2), a mitochondrial enzyme highly expressed in the liver, plays a major role in the acetaldehyde metabolism into nontoxic acetic acid [3]. In addition to ALDH2, ALDH class-1A1 (ALDH1A1), a cytosolic enzyme highly expressed in the liver, also contributes to the acetaldehyde metabolism [4]. ALDH class-3A1 (ALDH3A1), another cytosolic enzyme in the liver, might assist ALDH2 in the ethanol metabolism, even though it selectively oxidized aromatic and medium-chain aldehydes [5]. In Asian countries, more than 40% of people have the ALDH2 polymorphism that leads to the accumulation of acetaldehyde, not only causing serious vasodilation and facial flushing, but also increasing the risk of cancer development [6]. Therefore, it is quite plausible that the enhancement of the liver ALDH activities by intake of certain food ingredients could help prevent alcohol-intolerant individuals with a low ALDH activity from experiencing the alcohol-induced abnormal reaction, and thus chronic diseases.

Flavonoids, the major group of dietary polyphenols, are believed to contribute to the prevention of lifestyle-related diseases, such as metabolic syndrome, atherosclerosis, coronary heart disease, etc. [7,8]. Although the bioavailability of flavonoids plays a crucial role in their biological activity in vivo, most of the flavonoids in diets exist as their glycoside forms, only a limited amount of which can be absorbed in the upper gastrointestinal tract after metabolism [9]. The flavonoids unabsorbed in the small intestine, including their unmetabolized glycosides, as well as the metabolized glucuronides and sulfates, are deconjugated into their aglycone forms by bacterial enzymes in the colon, then further catabolized into ring fission products, such as phenolic acids [9,10]. In addition to the conjugate-type metabolites of flavonoids, the bacterial ring fission catabolites are also important determinants to evaluate the actual biological properties of flavonoids in vivo.

Quercetin, one of the most ubiquitous flavonol-type flavonoids in fruits and vegetables, is considered to contribute to the health maintenance of humans [11]. In general, quercetin occur in plants as glycosides, i.e., quercetin 3-glucoside (Q3G) is the most common quercetin glycoside and quercetin 4ʹ-glucoside (Q4ʹG) is one of the major quercetin glycosides in onions [7]. A previous study demonstrated that Q4ʹG has a strong reducing effect on lipid peroxidation in the rat intestinal mucosa, even though it has a much lower radical scavenging activity than Q3G [12]. This finding suggested that the effective bacterial metabolism of Q4ʹG in the intestinal mucosa is attributable to its in vivo antioxidant potential. A metabolic study using radiolabeled Q4ʹG [13] uncovered that, in the small intestine, most Q4ʹG was metabolized into conjugated quercetin, such as glucuronide, sulfate, and methylated derivatives, just trace amounts of which were excreted into the urine. However, the conjugated quercetin metabolites disappeared in the cecum and the colon because they were catabolized into phenolic acids, i.e., 3-hydroxyphenylacetic acid (OPAC) and 3,4-dihydroxyphenylacetic acid (DOPAC), mainly by the colonic microbiota [14]. The radiolabeled OPAC was identified as a major metabolite of the radiolabeled Q4ʹG in the feces, whereas substantial amounts of hippuric acid (HPA) and OPAC, along with a smaller amount of benzoic acid, were detected in the urine [14]. It is thus assumed that the first catabolite is DOPAC, which is subsequently dehydroxylated to form OPAC, followed by further catabolism into HPA or benzoic acid. OPAC was also detected as a major catabolite in the urine of human subjects after chocolate consumption [15]. Accordingly, OPAC has been identified as the main ring fission product of other quercetin glycosides, such as rutin [16] and hyperoside [17]. These findings strongly support the idea that OPAC is the most predominant and stable catabolite from quercetin glycosides in humans.

We have previously compared the effects of the major phenolic acid catabolites of Q4ʹG, including DOPAC, OPAC, and HPA, to evaluate their potential as antioxidants [18]. DOPAC was thus identified as the most potent phenolic acid derived from quercetin glycosides, as an inducer of drug metabolic enzymes, as well as a free radical scavenger [18]. DOPAC also significantly inhibits the hydrogen-peroxide-induced cytotoxicity in mouse hepatocytes independent of its radical scavenging effect. On the other hand, OPAC showed neither a radical scavenging effect nor significant potentiation of the gene expression of drug metabolic enzymes [18]. This is consistent with another report showing that OPAC is not able to inhibit the formation of advanced glycation end products and neuronal cell death [19]. To the best of our knowledge, the biological relevance of OPAC as an antioxidant or a cytoprotector has yet to be established. Quercetin has been reported to protect against alcohol-induced liver injury in the mouse model [20]. However, the involvement of quercetin catabolites in the regulation of the ALDH activity, and thus protection against acetaldehyde, is still unclear. The modulating effect of OPAC, the major ring fission product of quercetin glycosides, on the ALDH activity remained to be examined, even though DOPAC was reported to enhance the total ALDH activity [21]. In this study, we assessed OPAC as a unique type of ALDH activity enhancer derived from quercetin glycoside metabolism using the cultured hepatocyte models. Experiments using the compounds structurally related to OPAC were conducted to uncover the essential structure required for enhancement of the ALDH activity. We also demonstrated here that OPAC enhances the total ALDH activity by transcriptional regulation through the aryl hydrocarbon receptor (AhR)-dependent pathway. Furthermore, the pretreatment of OPAC significantly inhibited the acetaldehyde-induced cytotoxicity in an AhR-dependent and nuclear factor erythroid-2-related factor 2 (Nrf2)-independent manner.

## 2. Results

### 2.1. OPAC Enhanced the Total ALDH Enzyme Activity

**Figure 1:**
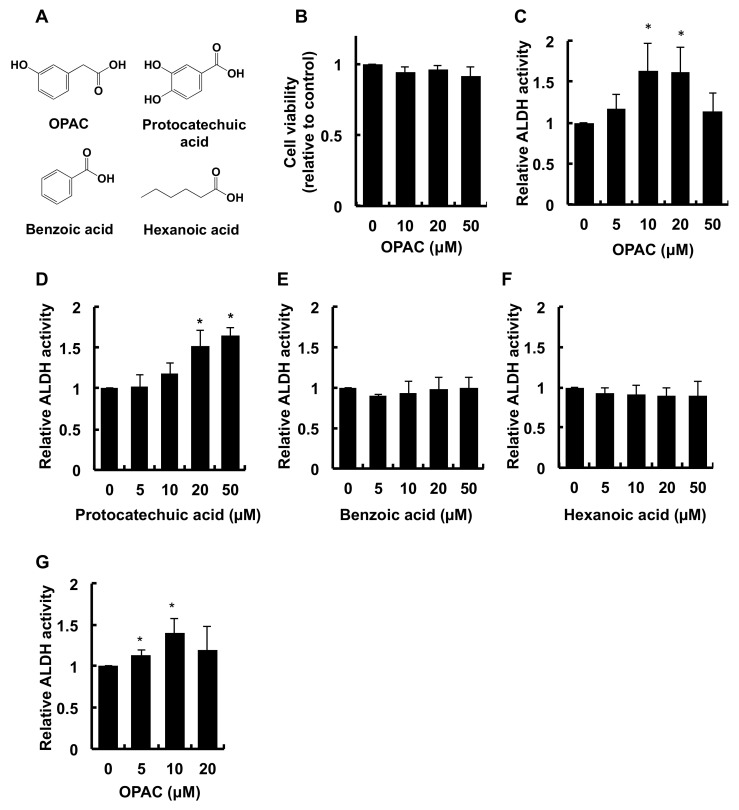
Enhancing effect of OPAC on the total ALDH activity in hepatoma cells. (**A**) Chemical structures of OPAC and its related compounds, such as protocatechuic acid, benzoic acid, and hexanoic acid. (**B**) Effect of OPAC on cell viability. Hepa1c1c7 cells were treated with the indicated concentrations of OPAC for 6 h. Cell viability was measured using an MTT assay. (**C**–**F**) Modulating effect of OPAC and its related compounds on the total ALDH activity in mouse Hepa1c1c7 cells. Hepa1c1c7 cells were treated with the indicated concentrations of OPAC (**C**), protocatechuic acid (**D**), benzoic acid (**E**), and hexanoic acid (**F**) for 6 h. The total ALDH activity was determined by a microplate reader (340 nm). The values are expressed as the ratio of the control pretreated only with DMSO. (**G**) Enhancing effect of OPAC on the total ALDH activity in human HepG2 cells. HepG2 cells were treated with the indicated concentrations of OPAC for 6 h. The total ALDH activity was determined by a microplate reader (340 nm). The values are expressed as the ratio of the control pretreated only with DMSO. All values are means ± SD of three independent experiments. *p < 0.05 vs. control.

Since we observed that OPAC (Figure 1A), even at the concentration of 50 μM, induced no significant cytotoxicity (Figure 1B), its maximum concentration to test was 50 μM. As shown in Figure 1C, the treatment of Hepa1c1c7 cells with OPAC for 6 h resulted in an increase in the total ALDH activity. The total ALDH activity in the cells treated with 10 μM and 20 μM OPAC was about 1.6-fold higher than that of the control, whereas no significant effect on the activity was observed at 50 μM. To clarify the structural requirement for the enhancement of the total ALDH activity, we next compared the modulating effects of the compounds structurally related to OPAC in Hepa1c1c7 cells. We previously reported that DOPAC, having a catechol (*ortho*-diphenol or *ortho*-dihydroxybenzyl) moiety, significantly enhanced the total ALDH activity [21]. Similarly, protocatechuic acid, having the same moiety (Figure 1A), dose-dependently potentiated the total ALDH activity (Figure 1D). On the other hand, benzoic acid, having a benzyl moiety without a hydroxy group, as well as hexanoic acid with an aliphatic hydrocarbon chain, showed no significant effect on the ALDH activity (Figure 1E,F). Furthermore, as shown in Figure 1G, OPAC also enhanced the total ALDH activity in human hepatocellular carcinoma HepG2 cells, even though the OPAC concentrations required for the potentiation between the cell lines were slightly different. These results suggested that OPAC is a potential enhancer of the total ALDH enzyme activity, not only in mice, but also in humans.

### 2.2. OPAC Enhanced the Protein and mRNA Levels of ALDH Isozymes

**Figure 2:**
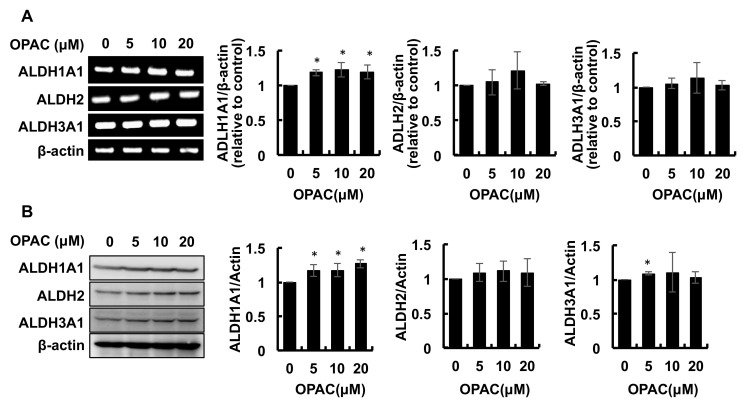
Modulating effects of OPAC on the gene and protein expressions of the classical family of ALDHs: ALDH1A1, ALDH2, and ALDH3A1. (**A**) The total RNA was extracted from the Hepa1c1c7 cells treated with OPAC for 6 h; then, an RT-PCR analysis for each gene was carried out. Representative blots and quantitative data for ADLH1A1, ADLH2, and ADLH3A1 are shown. (**B**) Hepa1c1c7 cells were treated with OPAC for 6 h, and the total cell lysates were subjected to a Western blot analysis. All values are expressed as means ± SD of three separate experiments. *p < 0.05 vs. control.

Among the 19 enzymes belonging to the human ALDH superfamily, ALDH1A1, ALDH2, and ALDH3A1 have been suggested to be responsible for acetaldehyde metabolism [3,4,5]. To further confirm the transcriptional regulation of ALDH isozymes by OPAC, we checked the mRNA levels of ALDH1A1, ALDH2, and ALDH3A1. As shown in Figure 2A, only the gene expression of ALDH1A1 was significantly increased by OPAC after a 6-h treatment. The concentration-dependent tendency of this isozyme was similar to that of the total activity. We next examined whether OPAC increased the protein expression of these ALDH isozymes. As shown in Figure 2B, the 6-h OPAC treatment significantly enhanced the protein levels of ALDH1A1 and ALDH3A1, but not that of ALDH2. The concentration-dependent tendency of the ALDH1A1 protein was also similar to that of the total activity.

### 2.3. OPAC Activated the AhR Pathways

**Figure 3:**
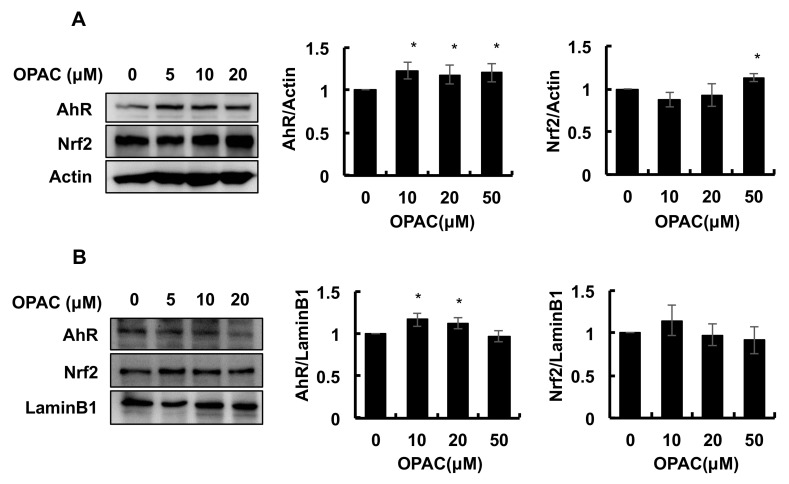
Modulating effects of OPAC on the total and nuclear levels of AhR and Nrf2 proteins. Hepa1c1c7 cells were treated with OPAC for 1 h, and the nuclear fractions, as well as the total cell lysates, were subjected to a Western blot analysis. (**A**) Effect of OPAC on the protein level of AhR and Nrf2 in the whole lysate. (**B**) Effect of OPAC on the nuclear accumulation of AhR and Nrf2. All values are expressed as means ± SD of three separate experiments. * *p* < 0.05 vs. control.

**Figure 4:**
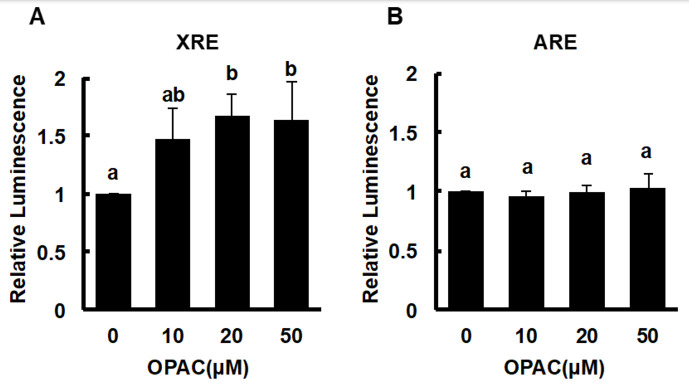
Modulating effects of OPAC on the transcriptional activities of AhR (**A**) and Nrf2 (**B**). HaCaT cells stably expressing the XRE- or ARE-reporter gene (XRE-NLuc::HaCaT or ARE-NLuc::HaCaT) were treated with OPAC for 6 h, and the reporter luciferase expression was measured as luminescence by a microplate luminometer. All values are expressed as means ± SD of three separate experiments. The different letters above the bars indicate significant differences among the treatments for each condition (*p* < 0.05).

Previous studies demonstrated that some organosulfur compounds, such as benzyl isothiocyanate and sulforaphane, increased the total ALDH activity through activation of the Nrf2-dependent pathway [22,23]. On the contrary, involvement of an AhR/xenobiotic response element axis in the gene expressions of several ALDH isozymes has been indicated [24,25]. Therefore, the modulating effect of OPAC on the nuclear translocation, as well as the protein expression of AhR and Nrf2 was examined. As shown in Figure 3A, the total level of the AhR protein was significantly increased by OPAC treatment for 1 h, whereas that of Nrf2 was not significantly changed at the concentrations required for enhancement of the total ALDH activity. Consistently, the nuclear level of AhR, but not Nrf2, was significantly enhanced by OPAC at the concentrations required for potentiation of the total ALDH activity (Figure 3B). We next checked the effect of OPAC on the transcriptional activities of AhR and Nrf2 by a luciferase assay using reporter genes having a xenobiotic response element (XRE) and an antioxidant response element (ARE), respectively. As shown in Figure 4A, 20 μM OPAC significantly increased the transcriptional activity of AhR, even though the cell line was different from that of the hepatocyte. In contrast, Nrf2 activity was not significantly changed by OPAC, even at 50 μM (Figure 4B). These results supported the idea that OPAC has the capability to activate AhR-dependent gene expression.

### 2.4. OPAC Protected the Cells from the Acetaldehyde-Induced Toxicity in an AhR-Dependent Manner

**Figure 5:**
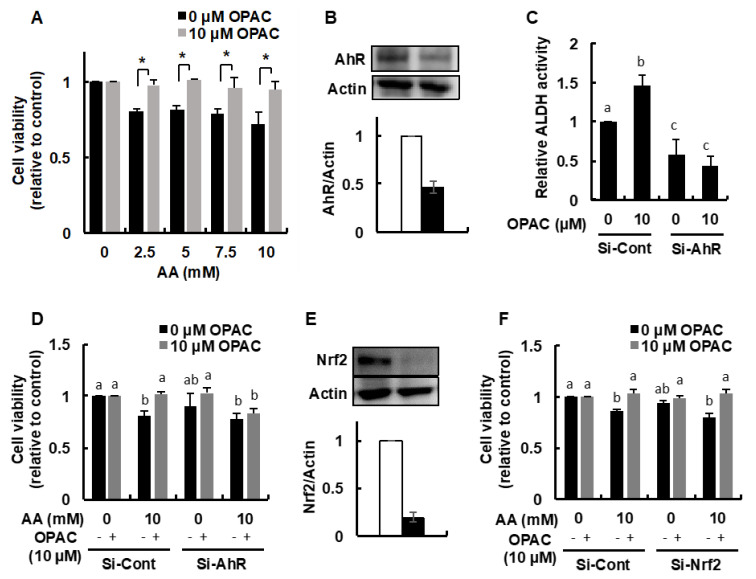
AhR-dependent protection against the acetaldehyde-induced cytotoxicity by OPAC. (**A**) Inhibitory effect of OPAC pretreatment on the acetaldehyde-induced cytotoxicity. After Hepa1c1c7 cells were pretreated with OPAC for 6 h, the cells were treated with different concentrations of acetaldehyde for 3 h; then, an MTT assay was carried out. (**B**) Modulating effect of AhR siRNA on the AhR protein level in the whole cell lysates. Hepa1c1c7 cells were transfected with the AhR siRNA or control scrambled siRNA, then incubated for 48 h. The total cell lysates were subjected to a Western blot analysis (*n* = 2). (**C**) Modulating effect of AhR siRNA on the total ALDH activity. The Hepa1c1c7 cells transfected with the AhR siRNA or control scrambled siRNA were treated with OPAC for 6 h, and the total ALDH activity was determined by a microplate reader (340 nm). (**D**) Impairment of the OPAC-enhanced resistance to the acetaldehyde-induced cytotoxicity by the AhR siRNA. After the Hepa1c1c7 cells transfected with siRNA were pretreated with OPAC for 6 h, the cells were treated with different concentrations of acetaldehyde for 3 h; then, an MTT assay was carried out. (**E**) Modulating effect of Nrf2 siRNA on the Nrf2 protein level in the whole cell lysates. Hepa1c1c7 cells were transfected with the Nrf2 siRNA or control scrambled siRNA, then incubated for 48 h. The total cell lysates were subjected to a Western blot analysis (*n* = 2). (**F**) No significant effect of the Nrf2 siRNA on the OPAC-enhanced resistance to the acetaldehyde-induced cytotoxicity. All values are expressed as means ± SD of three separate experiments and analyzed by a Student’s *t*-test or a one-way ANOVA, followed by Tukey’s HSD using XLSTAT software. The different letters above the bars indicate significant differences among the treatments for each condition (* *p* < 0.05 vs. control).

We further examined whether the acetaldehyde-induced cytotoxicity was inhibited by the OPAC pretreatment. We initially confirmed that the incubation of acetaldehyde at the concentrations from 2.5 to 10 mM for 3 h significantly decreased the cell viability (Figure 5A), consistent with previous reports [21,22]. The 6-h pretreatment of 10 μM OPAC, the minimum concentration required for enhancement of the total ALDH activity, completely rescued the cells from the acetaldehyde-induced cytotoxicity.

To examine whether AhR is involved in the OPAC-mediated protection against acetaldehyde, we used the siRNA technique to knockdown the AhR protein expression. The AhR siRNA transfection depleted the AhR level to 47% compared to the control (Figure 5B). Silencing AhR significantly decreased not only the basal ALDH activity to 57%, but also the OPAC-enhanced activity to a lower level than the control (Figure 5C). Even though the AhR siRNA has no cytotoxic effect on Hepa1c1c7 cells, it completely cancelled the protection against acetaldehyde by OPAC (Figure 5D). On the other hand, the Nrf2 siRNA, decreasing its protein level to 19% of the control (Figure 5E) as previously reported [22], did not influence the protective effect of OPAC on the acetaldehyde-induced cytotoxicity (Figure 5F).

## 3. Discussion

In the present study, we demonstrated the modulating effects of OPAC on the total ALDH activity in not only mouse hepatoma Hepa1c1c7 cells, but also human hepatoma HepG2 cells (Figure 1). The present results also suggested the protective potential of OPAC against the acetaldehyde-induced cytotoxicity (Figure 5A). Among the phenolic acid catabolites, DOPAC was previously identified as an enhancer of total ALDH activity [21]. The minimum concentration of OPAC required for this effect (10 μM, Figure 1C) was equivalent to that of DOPAC [21], suggesting that OPAC is another potential enhancer of the ALDH activity derived from the quercetin bacterial metabolism, which contributes to the same extent as DOPAC. DOPAC is believed to be the first ring fission product in the gut bacterial metabolism of quercetin, and OPAC is produced from DOPAC by dehydroxylation [9,14]. Alternatively, OPAC was detectable in the liver, urine, and feces of the rat fed quercetin glucoside, whereas DOPAC was not [8]. Therefore, OPAC is expected as a more stable and less metabolizable metabolite of quercetin than DOPAC, capable of circulating in the peripheral circulation and being distributed to multiple organs without modification.

To identify the structural factor required to potentiate the total ALDH activity, we conducted a structure–activity relationship study using the compounds structurally related to OPAC in Hepa1c1c7 cells. This study showed that OPAC, as well as protocatechuic acid, having a phenol group, significantly enhanced the total ALDH activity in Hepa1c1c7 cells (Figure 1C,D). On the other hand, benzoic acid, having no hydroxy group in its benzene ring, as well as hexanoic acid, showed no enhancing effect. These results suggested that a benzene ring with at least one hydroxy group (monophenol group) is a prerequisite for the potentiation of the total ALDH activity.

The OPAC treatments significantly increased the gene and protein expressions of cytosolic ALDH1A1 (Figure 2). These results indicated that OPAC potentiates the total ALDH activity, possibly through a transcriptional regulation of these isozymes. Nrf2 is one of the key transcriptional factors which induces the expression of various xenobiotics-metabolizing genes, including ALDHs, that contain ARE in their promoter. We have confirmed that benzyl isothiocyanate, an organosulfur compound mainly derived from cruciferous vegetables, enhanced the total ALDH activity, as well as the gene expression of several ALDH isozymes, at least partly, through the Nrf2-dependent pathway [22]. In addition to Nrf2, AhR can regulate the gene expression of ALDH1A7, ALDH1B1, and ALDH3A1 [24,25]. OPAC increased the nuclear translocation of AhR (Figure 3B) and the transcriptional activity of AhR, determined by a luciferase assay (Figure 4). Furthermore, pretreatment of the AhR siRNA decreased both the basal and OPAC-enhanced ALDH activities (Figure 5C), suggesting that the total ALDH activity, at least partly, is regulated by the AhR-dependent pathway. Since ALDH3A1 was not drastically enhanced by OPAC (Figure 2) and the gene expression of ALDH1A1 was independent of AhR [25], other ALDH isozymes might be involved in the AhR-dependent total ALDH activity enhancement by OPAC. Even though other signaling molecules, such as STAT3 [26], proliferator activated receptors [27], and retinoic acid receptors [28], might also be involved in the regulation of the ALDH gene expression, AhR is one of the key transcriptional factors for the OPAC-enhanced ALDH activity. Moreover, AhR siRNA completely counteracted the protection against acetaldehyde by OPAC, but not for Nrf2 (Figure 5D,F). These results strongly suggested that OPAC mitigates the acetaldehyde-induced cytotoxicity, possibly through regulation of the total ALDH activity in an AhR-dependent and Nrf2-independent manner.

Acetaldehyde-induced cytotoxicity has been reported in a variety of cellular models, including the liver [29], large intestine [30], and neuron [31]. In addition to the electrophilic nature of acetaldehyde contributing to DNA and protein damage, reactive oxygen species produced via mitochondrial damage are also involved in its toxic mechanisms [32]. It is thus noted that not only the enhanced metabolism of acetaldehyde, but also the induction of the antioxidative defense system is a quite plausible mechanism for protection against acetaldehyde. Indeed, DOPAC inhibits both the hydrogen-peroxide- and acetaldehyde-induced cytotoxicity in mouse hepatocytes, possibly through the enhanced gene expression of antioxidant enzymes, as well as ALDHs [18,21]. On the other hand, we have previously indicated that OPAC is neither a radical scavenger (chemical antioxidant) nor an inducer of antioxidant enzymes (biologically active antioxidant), such as heme oxygenase 1 and glutamate-cysteine ligase catalytic subunit, in Hepa1c1c7 cells [18]. Since these enzymes are mainly regulated by the Nrf2-dependent pathway, it is suggested that Nrf2 is not involved in the regulation of the ALDH gene expressions by OPAC. Furthermore, induction of the antioxidative defense system could be ruled out in the mechanism underlying the OPAC-induced protection against acetaldehyde. This characteristic of OPAC neither acting as an antioxidant nor activating the Nrf2 pathway is very unique among polyphenols, most of which are not only potent chemical antioxidants, but also biologically active antioxidants inducing the antioxidative enzyme genes in an Nrf2-dependent manner [33].

OPAC effectively inhibited the acetaldehyde-induced cytotoxicity in the hepatocyte model. The cultured hepatocyte models using the hepatoma cells have several limitations. First, the most common drawback of this model is that it does not reflect the characteristics of hepatocytes in vivo because they are cancerous cells. Second, the acetaldehyde-induced cytotoxicity model is acute and transient, which is different from the sustained injury progression in continuous alcohol drinking. Third, since the ALDH2 gene is wild-type in this model, it is unclear whether OPAC would have the same effect in the cells having ALDH polymorphism with reduced activity. In addition, the OPAC concentrations required for enhancement of ALDH activity (~10 μM) might be supraphysiological, since a previous intervention study revealed that OPAC concentration in the plasma was achieved to approximately 100~400 nM after the consumption of berries (160 g/day) [34]. Future studies will be concerned with the in vivo significance of protection against liver injury by OPAC in several rodent models, as well as further understanding the signaling pathways required for upregulation of ALDHs.

In conclusion, we identified OPAC, a major intestinal catabolite of quercetin glycosides, as a different type of an enhancer of the total ALDH activity from DOPAC. OPAC may contribute not only to the potentiation of acetaldehyde metabolism by ALDHs, but also to protection from alcohol-induced liver injury after the ingestion of quercetin-rich diets. OPAC has more advantages for application as a food chemical than DOPAC or the parent quercetin because of its lower cytotoxicity and high resistance against metabolism.

## 4. Materials and Methods

### 4.1. Chemicals and Antibodies

OPAC, protocatechuic acid, and hexanoic acid were purchased from Sigma-Aldrich (St. Louis, MO, USA). Fetal bovine serum (FBS) was purchased from Nichirei Corporation (Tokyo, Japan). ReverTra Ace was purchased from TOYOBO Co., Ltd. (Osaka, Japan). Taq polymerase was purchased from Takara Bio Inc. (Kusatsu, Japan). Bio-Rad Protein Assay was purchased from Bio-Rad Laboratories (Hercules, CA, USA). Trizol reagent, α-minimum essential medium (α-MEM), and Pierce^TM^ BCA Protein Assay Kit were purchased from Thermo Scientific (Meridian Rd., Rockford. USA). Chemi-lumi One Super was purchased from Nacalai Tesque Inc. (Kyoto, Japan). Antibodies against ALDH2, AhR, Nrf2, lamin B1, actin, siRNA for Nrf2 (Catalog No. sc-37049), siRNA for AhR (Catalog No. sc-29655), and control scrambled siRNA (Catalog No. sc-37007) were purchased from Santa Cruz Biotechnology (Santa Cruz, CA, USA). Antibody against ALDH1A1 was purchased from Cell Signaling Technology (Beverly, MA, USA), and antibody against ALDH3A1 was obtained from Abcam (Cambridge, MA, USA). β-Nicotinamide-adenine dinucleotide, oxidized form (NAD^+^) was purchased from Oriental Yeast Co., Ltd. (Tokyo, Japan). All other chemicals were obtained from FUJIFILM Wako Pure Chemical Industries (Osaka, Japan).

### 4.2. Cell Culture

Hepa1c1c7 cells and HepG2 cells were obtained from the American Type Culture Collection (Manassas, VA, USA). The cells were maintained in α-MEM supplemented with 10% FBS, 4 mM L-glutamine, 100 U/mL penicillin, and 100 µg/mL streptomycin. The cells were grown at 37 °C in an atmosphere of 95% O_2_ and 5% CO_2_.

### 4.3. MTT Assay

Hepa1c1c7 cells (2 × 10^4^) were seeded in a 96-well plate. After the overnight preculture, the cells were treated with OPAC for 6 h. For the acetaldehyde-induced cytotoxicity, the cells were pretreated with OPAC for 6 h, followed by exposure to acetaldehyde at the indicated concentrations for 3 h. The cells were washed once with phosphate-buffered saline (PBS), then incubated with 0.5 mg/mL of an MTT solution for 2 h. The insoluble formazan precipitates were dissolved in DMSO, then the absorbance was measured at 570 nm by a microplate reader (Benchmarkplus, Bio-Rad Laboratories, Hercules, CA, USA). The cell viability values were expressed as the percentages over the corresponding controls.

### 4.4. ALDH Activity Assay

Hepa1c1c7 cells or HepG2 cells were treated with the indicated concentrations of the test compounds for 6 h. The ALDH activity was measured as previously described [21,22]. Briefly, the cell lysates were prepared by 1 mL lysis buffer (25 mM EDTA, 50 mM Tris (pH 8), 1 mM phenylmethylsulfonyl fluoride, 5 mM β-mercaptoethanol, and 0.1% sarcosyl). Then, 20 µL of 5 mM NAD^+^ and 20 µL of 5 mM propionaldehyde (substrate) were mixed with 200 µL of the total lysate. NADH was measured by the change in absorbance at 340 nm over 5 min. One unit was defined as the amount of enzyme activity that converts 1 μmol NAD^+^ to NADH per minute.

### 4.5. RT-PCR

Hepa1c1c7 cells were treated with OPAC at the indicated concentrations for 6 h. Total RNA was extracted with TRIzol reagent according to the manufacturer’s manual. The total RNA (5 µg) was reverse transcribed to cDNA using ReverTra Ace. The cDNA was used for PCR amplification with Taq polymerase. Primers used in PCR amplification were as follows: mALDH1A1, (F) 5′-gACAggCTTTCCAgATTggCTC-3′ and (R) 5′-AAgACTTTCCCACCATTgAgTgC-3′; mALDH2, (F) 5′-TgAAgACggTTACTgTCAAAgTgC-3′ and (R) 5′-AgTgTgTgTggCggTTTTTCTC-3′; mALDH3A1, (F) 5′-gATgCCCATTgTgTgTgTTCg-3′ and (R) 5′-CCACCgCTTgATgTCTCTgC-3′; mβ-actin, (F) 5′-gTCACCCACACTgTgCCCATCTA-3′ and (R) 5′-gCAATgCCAgggTACATggTggT-3′. The PCR conditions, including cycles and annealing temperatures, were optimized as follows: mALDH1A1, 20 cycles, 57 °C; mALDH2, 20 cycles, 57 °C; mALDH3A1, 25 cycles, 56 °C; mβ-actin, 16 cycles, 65 °C. The amplified PCR products were separated on an agarose gel (2%), stained with ethidium bromide, and visualized using an LAS3000 image analyzer (FujiFilm, Tokyo, Japan). The relative densities of bands were measured using Image J Software Program.

### 4.6. Western Blot Analysis

Hepac1c1c7 cells were treated with OPAC at the indicated concentrations for 1 h. After harvesting, the whole cell lysates and nuclear fraction were prepared as previously reported [22]. The whole cell lysates containing 30 μg of protein and the nuclear fractions containing 10 μg of protein were separated by SDS-PAGE. The separated proteins were transferred electrophoretically to PVDF membranes. The membranes were incubated with the antibodies for Nrf2 and AhR and the secondary antibody conjugated to horseradish peroxidase, then visualized by a Chem-Lumi One Super using an LAS3000 image analyzer (FujiFilm, Tokyo, Japan). Image J Software Program was used to measure the relative densities of bands.

### 4.7. Establishment of the Cell Line Stably Expressing the NanoLuc Reporter Gene

The cell lines stably expressing the reporter genes were established as previously reported [35]. Human HaCaT cells, obtained from the German Cancer Research Center (DKFZ, Heidelberg, Germany), were maintained in Dulbecco’s modified Eagle medium supplemented with 10% FBS. The cells were transfected with pNL(NlucP/XRE/Hygro) (#CS CS186808, Promega Japan, Tokyo, Japan) or pNL(NlucP/ARE/Hygro) (#CS180902, Promega). The cells stably expressing the transgenes were selected using hygromycin; then, the established cell lines were referred to as XRE-NLuc::HaCaT and ARE-NLuc::HaCaT.

### 4.8. Luciferase Assay

Luciferase assay using the established cell lines was performed as previously reported [35]. Briefly, XRE-NLuc::HaCaT cells or ARE-NLuc::HaCaT cells (1 × 10^4^) were plated in a 96-well white plate. After the 24-h preculture, treatment with OPAC at the indicated concentrations was carried out for 6 h. Then, 10 μL of the NLuc substrate (#N1120, Promega) was added to the cells. After the 5-min incubation, luminescence was measured using a microplate luminometer (SH-9000Lab, CORONA ELECTRIC, Hitachinaka, Japan). The reporter expression measured as luminescence was corrected based on the number of viable cells obtained as the absorbance.

### 4.9. RNA Interference

Hepa1c1c7 cells (2.0 × 10^5^) were seeded in a 6-well plate and transfected with si-control (Catalog No. sc-37007 from Santa Cruz, 120 μM), si-AhR (Catalog No. sc-29655 from Santa Cruz, 120 μM), and si-Nrf2 (Catalog No. sc-37049 from Santa Cruz, 120 μM) using Lipofectamine 3000 (Invitrogen, Waltham, MA, USA) according to the manufacturer’s instructions for 48 h. The transfected cells were treated with DMSO or 10 μM OPAC for 6 h, followed by MTT assay and ALDH activity assay.

### 4.10. Statistical Analyses

All the values are expressed as the mean of at least three independent experiments ± standard deviation. Statistical significance was determined by Student’s paired two-tailed t-test or one-way analysis of variance (ANOVA) followed by Tukey’s honestly significant difference (HSD) test using XLSTAT software (Addinsoft Inc., NewYork, NY, USA). A level of *p*-value < 0.05 was considered significant for all the comparisons.

## Data Availability

The data presented in this study are available from the corresponding author upon reasonable request.

## Abbreviations

Q3G, quercetin 3-glucoside; Q4ʹG, quercetin 4ʹ-glucoside; OPAC, 3-hydroxyphenylacetic acid; DOPAC, 3,4-dihydroxyphenylacetic acid; HPA, hippuric acid; ALDH, aldehyde dehydrogenase; ALDH2, ALDH class-2; ALDH1A1, ALDH class-1A1; ALDH3A1, ALDH class-3A1; AhR, hydrocarbon receptor; Nrf2, nuclear factor erythroid 2-related factor 2; XRE, xenobiotic response element; ARE, antioxidant response element.
